# Synovial Sarcoma of the Kidney: Diagnostic Pitfalls in a Case with Myxoid Monophasic Differentiation and No Epithelial Biomarkers Expression

**DOI:** 10.3390/ijms25137382

**Published:** 2024-07-05

**Authors:** Francesca Pagliuca, Emma Carraturo, Anna De Chiara, Silvia Vallese, Isabella Giovannoni, Rita Alaggio, Lucia Cannella, Salvatore Tafuto, Renato Franco

**Affiliations:** 1 Pathology Unit, Vanvitelli University Hospital, 80138 Naples, Italy; 2 Histopathology of Lymphomas and Sarcomas SSD, Istituto Nazionale Tumori-IRCCS-Fondazione “G. Pascale”, 80131 Naples, Italy; 3 Pathology Unit, Bambino Gesù Children’s Hospital, IRCCS, 00165 Rome, Italy; 4 S.C. Sarcomas and Rare Tumors, Istituto Nazionale Tumori-IRCCS-Fondazione “G. Pascale”, 80131 Naples, Italy; 5 Department of Mental and Physical Health and Preventive Medicine, University of Campania “Luigi Vanvitelli”, 80138 Naples, Italy

**Keywords:** synovial sarcoma, clear cell sarcoma of the kidney, *BCOR*, soft tissue tumours, RNA sequencing

## Abstract

Synovial sarcomas are soft tissue tumours of uncertain origin, most commonly found in the upper or lower extremities. They are characterised by distinctive chromosomal rearrangements involving the gene *SS18*. Synovial sarcomas can occasionally arise also in visceral sites, but retroperitoneal SSs are very unusual. Among them, a few primary renal synovial sarcomas have been described in the scientific literature. Primary renal synovial sarcomas tend to be monophasic and often show cystic changes. Histologically, they can closely resemble other primary kidney tumours, mainly paediatric tumours such as nephroblastoma and clear cell sarcoma of the kidney. In the current work, a primary synovial sarcoma of the kidney with unusual morphological features (extensively myxoid stroma and immunohistochemical positivity for BCOR) is described. Molecular analysis, through targeted RNA sequencing, was of invaluable help in reaching the correct diagnosis. Despite locally advanced disease at presentation, the patient showed an unexpectedly brilliant response to chemotherapy.

## 1. Introduction

Synovial sarcoma (SS) is a malignant soft tissue neoplasm included among tumours of uncertain origin in the latest WHO classification [1]. It is associated with a pathognomonic chromosomal translocation t(X;18)(p11;q11), involving the gene *SS18* on chromosome 18 and either *SSX1*, *SSX2* or *SSX4* on chromosome X as a fusion partner [1]. SS is typically found in the upper or lower extremities of young patients, although it can virtually arise in any body location, including the head and neck, the abdomen and the retroperitoneum [1]. Histologically, SSs present as monomorphic spindle cell sarcomas with variable epithelial differentiation, classified as biphasic (both the epithelial and spindle cell components) or monophasic (one of the components, usually the spindle cell component) [1,2]. The epithelial component, when present, consists of cuboidal or columnar cells with moderate amounts of eosinophilic cytoplasm, arranged in glandular (or papillary/alveolar) structures [1]. The characteristic spindle cell component is always found, consisting of small cells with hyperchromatic nuclei and scant cytoplasm [1,2]. Poorly differentiated areas, characterised by nuclear atypia, increased cellularity and high mitotic activity (>6 mitoses/mm^2^ or >10 mitoses per 10 high-power fields) can be found in both biphasic and monophasic SS and may even predominate, especially in older patients [1,2]. The stroma in SS is usually scant and collagenic, with haemangiopericytoma-like vessels and scattered mast cells. Myxoid change, areas of calcification and/or ossification can be focally seen [1,2]. The immunohistochemical profile is largely unspecific [2]. SSs in most cases express CD99, CD56, and Bcl2 (which may show membranous staining as seen in Ewing sarcoma). The epithelial component of SS variably expresses cytokeratins (CKs) while EMA is more widely expressed, at least focally in spindle cells and poorly differentiated areas [1]. Focal S100 expression may be detectable in as many as 40% of SSs [1]. Alpha-smooth muscle actin is positive in less than half of tumours; desmin is rarely positive but caldesmon is consistently negative [1]. TLE1 transcriptional corepressor immunostaining is found in the majority of the cases and shows moderate to strong nuclear staining. It is considered a relatively sensitive and specific marker for SS, although it may also be expressed by other soft tissue tumours that enter in differential diagnosis with SS (solitary fibrous tumour, malignant peripheral nerve sheath tumour) [1].

Primary renal SSs are very rare [1], with few cases described in the literature (Table 1). SSs arising in the kidney may histologically mimic other types of primary renal neoplasms, including paediatric tumours such as clear cell sarcoma of the kidney (CCSK). The occurrence of uncommon histological features in SS, like extensive myxoid stromal change, can be confounding and hinder its correct recognition.

Molecular analysis, through techniques like fluorescent in situ hybridization (FISH) and RNA sequencing, is crucial to solve the diagnosis in these cases.

We present a case of renal myxoid SS, discussing the potential diagnostic pitfalls of myxoid SS arising from the kidney.

## 2. Case Report

In April 2023, a 37-year-old male patient with an unremarkable past medical history and no family history of cancer was referred to the Emergency Department in a state of hypovolemic shock with profuse sweating and acute abdominal pain.

He complained of macroscopic haematuria in the previous hours and reported a similar event having occurred approximately two months before.

Laboratory tests showed normal renal function and a slight reduction in haemoglobin values (11.5 g/dL). The introduction of a bladder catheter confirmed the presence of haematuria.

**Figure 1:**
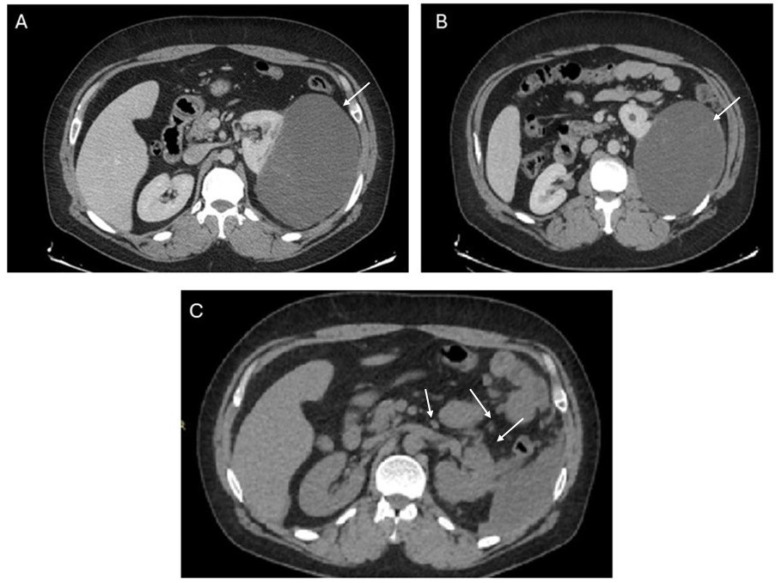
Imaging findings. (**A**,**B**) Pre-operative CT scans: exophytic, homogenous mass on the left kidney (arrow); (**C**) post-operative CT scan showing the presence of solid abdominal and peri-renal implants (arrows).

CT scan revealed the presence of a large left renal mass of 150 × 102 mm, classified as a Bosniak IV cyst on computed tomography (CT) according to the Bosniak classification system of renal cystic masses (Figure 1A,B). A kidney-sparing enucleation of the mass was performed in the suspicion of a primary haemorrhagic renal lesion. Conservative surgery was chosen due to the young age of the patient and as indicated by ESMO guidelines for renal tumours.

**Figure 2:**
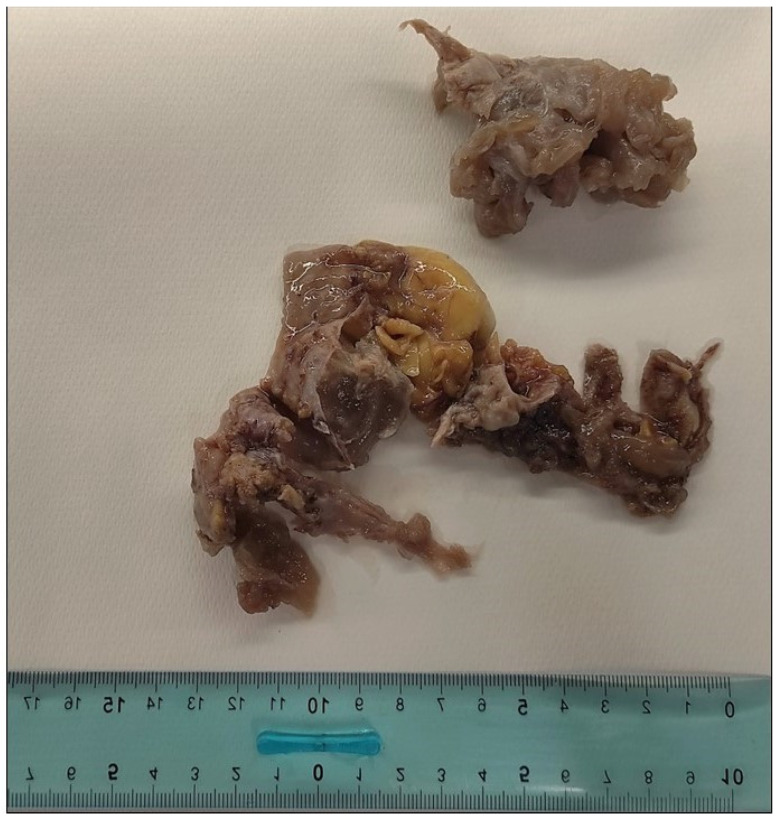
Gross findings: plurifragmented cystic sample with gelatinous areas.

Grossly, the mass appeared as a multiloculated myxoid cyst (Figure 2).

**Figure 3:**
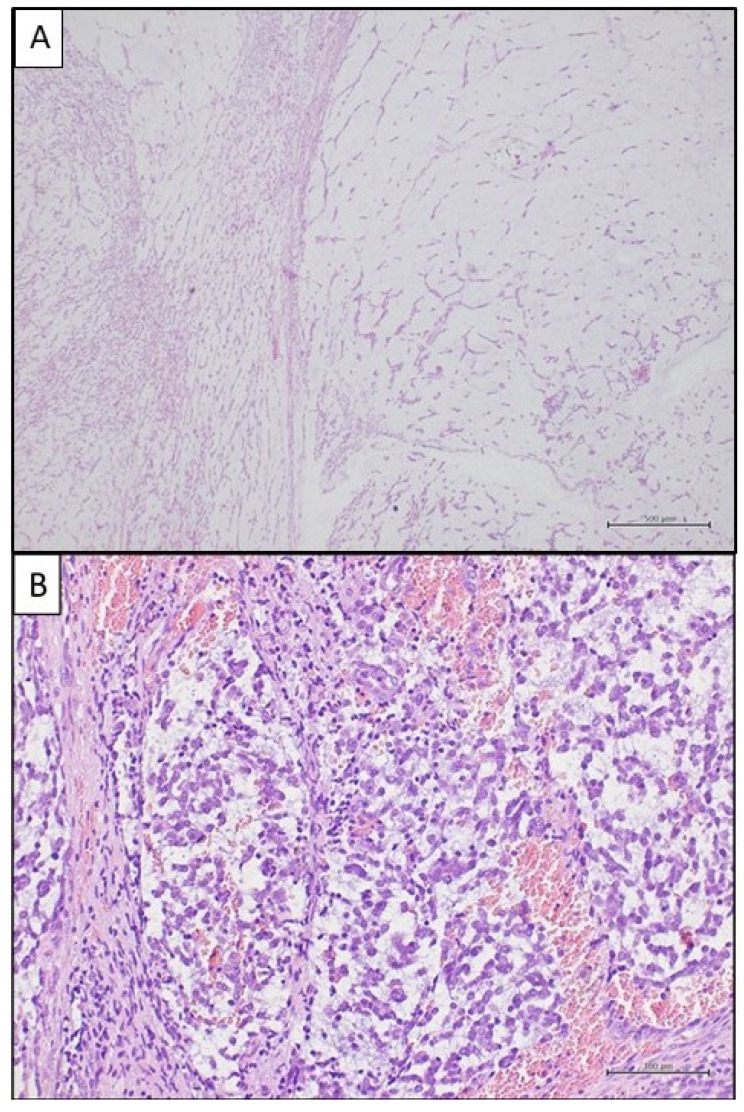
Histological features: (**A**) the tumour is mainly composed of large hypocellular myxoid areas (on the right), with scattered areas of increased cellularity (on the left): haematoxylin and eosin stain; original magnification: 40×. (**B**) At higher magnification, neoplastic cells appear ovoid-to-spindled, with scant cytoplasms and hyperchromatic nuclei: haematoxylin and eosin stain; original magnification: 200×.

Histologic examination showed a neoplastic proliferation of small blue round-to-spindled cells in an abundant myxoid matrix, arranged in a solid and fascicular pattern of growth (Figure 3A,B). Myxoid hypocellular areas predominated. The cells had moderately pleomorphic, ovoid-to-fusiform nuclei, with coarse chromatin, scant cytoplasm and a mitotic index of eleven mitoses per ten high-power fields. Necrosis was not seen.

The immunohistochemical study highlighted strong positivity for PAX-8, CD56, vimentin, WT-1 (cytoplasmic), cyclin D1, Bcl-2 (cytoplasmic), and INI-1. Negative immunostains included CK AE1/AE3, CK8/18, high molecular weight CKs, CK7, CK20, EMA, chromogranin, synaptophysin, calretinin, S100, HMB45, CD10, racemase, CD34, CD99, desmin, alpha-smooth muscle actin, muscle-specific actin, calponin, BRAF V600E.

Taking into account the tumour morphology and the extensively myxoid neoplastic stroma, the following hypotheses were mainly considered in the differential diagnosis: extraskeletal myxoid chondrosarcoma and CCSK. The former was ruled out as FISH analysis excluded the presence of *NR4A3* and *EWSR1* gene rearrangements.

**Figure 4:**
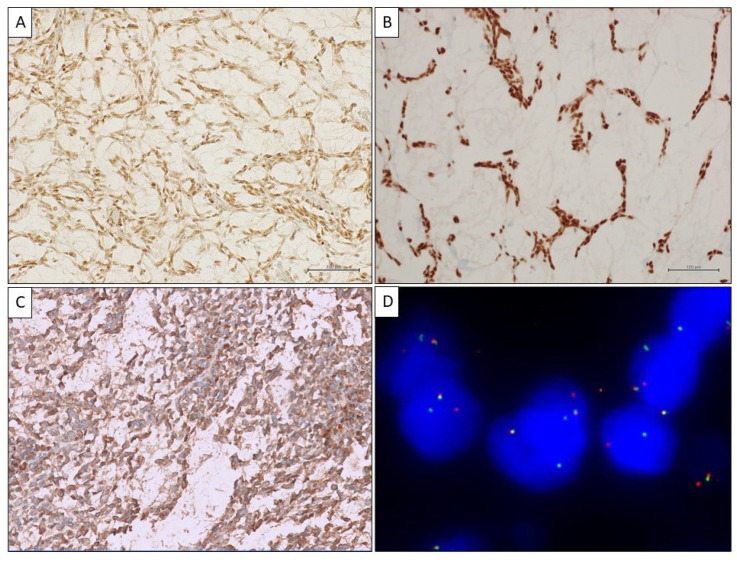
(**A**–**C**) Immunohistochemical stains. (**A**) Positivity for TLE1 (original magnification: 200×); (**B**) positivity for SS18-SSX immunohistochemistry (original magnification: 200×); (**C**) aberrant positivity for BCOR (original magnification: 200×). (**D**); FISH ZytoLight SPEC SS18 Dual Color Break Apart Probe: SS18(18q11.2) showing the presence of SYT rearrangement.

Interestingly, immunohistochemical cytoplasmic positivity for BCOR was seen (Figure 4C) and cyclin D1 was also positive, corroborating CCSK as a diagnostic option, in spite of the patient’s adult age.

**Figure 5:**
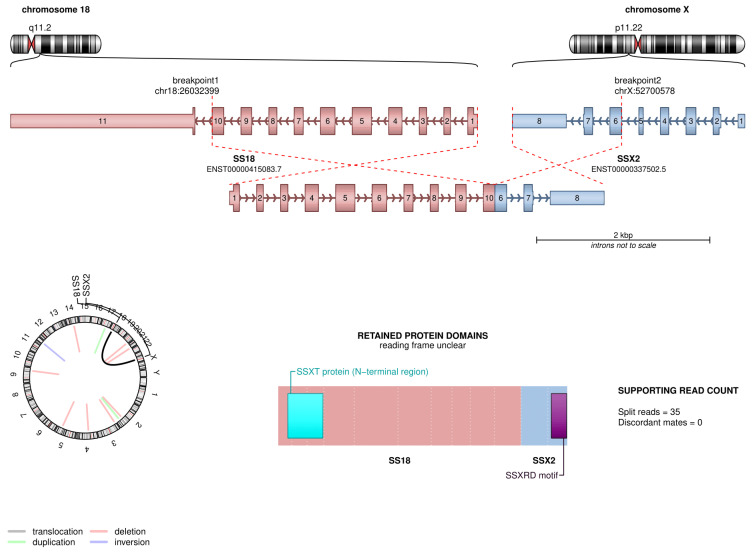
RNA sequencing results.

In order to identify genomic rearrangements, a transcriptomic analysis (RNA sequencing) was carried out. Unexpectedly, RNA sequencing showed the presence of an *SS18::SSX2* (exon 10::exon 6) fusion transcript, leading to a final diagnosis of monophasic SS (Figure 5). On the other hand, no BCOR rearrangements were detected.

FISH analysis was further performed to confirm NGS data, confirming the presence of *SYT* rearrangement (Figure 4D). 

TLE1 and SS18-SSX immunohistochemical stains were subsequently ordered and resulted positive in neoplastic cells (Figure 4A,B).

Post-operative CT scan showed the presence of disseminated disease in the abdomen with several retroperitoneal masses, solid residual tumour tissue on the kidney and peritoneal carcinosis (Figure 1C).

First-line chemotherapy with epirubicin 60 mg/mq days 1 and 2 and ifosfamide 3000 mg/mq days 1, 2 and 3 every 3 weeks was started and after three cycles an unusual partial response was achieved with reduction of all the abdominal lesions and small residual retroperitoneal disease.

The patient reported only mild haematologic toxicity and was on GCSF prophylaxis for seven days starting 6 days after chemotherapy. After other three cycles, a further decrease in the size of all the lesions was registered.

In order to maintain the optimal response achieved, three cycles with ifosfamide 3000 mg/die days 1, 2 and 3 every 3 weeks in monotherapy were administered.

To date (June 2024), the patient is in follow-up without macroscopic evidence of disease at CT scan.

## 3. Materials and Methods

For RNA sequencing analysis, RNA was extracted from FFPE tumour tissue using Maxwell CSC instrument (Promega, Madison, WI, USA) with the Maxwell RSC RNA FFPE kit (Promega, Madison, WI, USA) according to the manufacturer’s protocol. Total RNA was used a targeted RNA-Seq with SureSelectXT HS2 RNA system with Human All Exon V6 + COSMIC Probe (Agilent Technologies, Santa Clara, CA, USA) was used according to the manufacturer’s instructions (version A1, September 2020). The sequencing run was performed in paired-end mode (2 × 151-bp reads) using the Illumina NextSeQ 550 platform (Illumina, San Diego, CA, USA) and the data were analysed as described previously [79]. For the evaluation of the *SS18* gene rearrangement by break-apart FISH assay, three 4 μm-thick sections were cut from each formalin-fixed paraffin-embedded (FFPE) sample and subjected to FISH using the BOND FISH kit (Leica Biosystems, Newcastle Upon Tyne, UK) on an automated BOND system (Leica Biosystems). A formamide mixture is included in this kit to lessen nonspecific hybridization of nucleic acid probes. The ZytoLight SPEC SS18 Dual Color Break Apart Probe (ZytoVision, Bremerhaven, Germany) was used specifically to identify *SYT* rearrangement. Using an automated CytoVision platform (Leica Biosystems), slides were counterstained with 4′,6- diamidino-2-phenylindole dihydrochloride (DAPI) in antifade solution. With the Leica DM5500 B automated fluorescent microscope (Leica Biosystems), FISH interpretation was carried out using the ET-D/O/G filter for double Spectrum Green plus Spectrum Orange. FISH signals were detected in a minimum of 100 non-overlapping intact nuclei.

## 4. Discussion

SS is considered a tumour of uncertain derivation and accounts for 5–10% of all soft tissue sarcomas [1].

It may occur at any age, mainly in young patients (peak incidence: third decade) [2], with no clear gender predilection. It can arise anywhere, but it most commonly affects the lower or upper extremities, often close to a joint [1,2].

Despite its tendency to arise in proximity to articular structures, the name “synovial sarcoma” is actually a misnomer, as there is no evidence of derivation from the synovia [2,80]. The cell of origin of SS has been long discussed and it is still obscure: SS is probably derived from a multipotent mesenchymal stem cell [81] or from immature myoblasts [80]. The intra-abdominal location is utterly uncommon for SS.

The characteristic and diagnostic molecular alteration in SS is the *SS18::SSX1/2/4* fusion gene, in which *SS18* on chromosome 18 is fused to *SSX* genes on the X chromosome. The fusion partner for *SS18* is *SSX1* in the majority of cases (approximately 70%), followed by *SSX2* (approximately 30%) while fusions involving *SSX4* are only rarely encountered [2,81]. Usually, *SS18::SSX* fusions show the same intronic breakpoints; nevertheless, some unusual variants and cryptic rearrangements have been sporadically reported. *SS18* encodes for a component of the mSWI/SNF chromatin remodelling complex, ubiquitously expressed in normal human tissues [82]. On the other hand, *SSX* genes encode for histone-binding proteins whose expression has been observed, under normal conditions, only in spermatogonia and in thyroid tissue. The oncogenic effect of *SSX18::SSX* fusion proteins has been elucidated in recent years [82,83]. The oncogenic protein replaces wild-type SS18 in the mSWI/SNF (BAF) chromatin remodelling complexes, thus displacing the BAF47 subunit and interfering with their gene-activating functions [83]. As a result, the altered BAF complexes drive aberrant activation of transcription factors such as MYC, SOX2, PAX3, and PAX7 [82,83].

The clinical implications and prognostic significance, if any, of the type of fusion gene in SS are still a matter of debate [84]. Some studies, including retrospective multi-institutional studies, have observed that patients with *SS18::SSX2* show an overall better prognosis, with better overall survival, compared with patients with *SS18::SSX1* [84,85,86]. However, other studies have failed to find any prognostic difference based on the fusion variant [87,88].

Whatever the *SS18::SSX* variant involved, the presence of the translocation should be detected, either by FISH or reverse transcription polymerase chain reaction (RT-PCR), to confirm a diagnosis of SS [2,81].

The prognosis of SS is variable: tumour size and stage, the extent of poorly differentiated areas and tumour grade have prognostic relevance. Overall, the outcome is better for paediatric patients and for extremity-based, small (<5 cm in diameter) tumours, with a mitotic index < 6 mitoses/mm^2^ [2].

The treatment of choice for SS is complete surgical resection with tumour-free margins [81]. Adjuvant or neoadjuvant radiation therapy and chemotherapy are usually restricted to patients with high-risk tumours or in cases of metastatic or unresectable disease [81,89]. The most commonly administered regimen is a combination of ifosfamide and doxorubicin and the most effective responses are usually seen in younger patients [89].

While focal myxoid change in SS is a frequently encountered feature, SS may very unusually be extensively myxoid, mimicking other soft tissue tumours [2]. The occurrence of myxoid SS is rare but has been described. Krane et al. report a series of seven myxoid SSs, four arising in the lower extremities, two in the upper extremities and one in the head and neck region [90]. The median patient age was 20 years [90]. Histologically, five cases were monophasic and two had biphasic morphology [90]. All those cases had areas with more typical SS features, such as stromal mast cells, a fascicular growth pattern with a variable collagenised stroma and a haemangiopericytoma-like vascular pattern [90]. In addition, all cases were focally positive for EMA and most of them showed focal positivity to cytokeratins [90].

Few other cases of myxoid SS have been published in the scientific literature, all involving the hand or foot [91,92,93].

To the best of our knowledge, ours is the first case of myxoid SS arising in the kidney. Features that delayed the correct diagnosis were the striking stromal myxoid changes and the total absence of immunohistochemical expression of epithelial markers.

The kidney is an unusual location for SS; presumably, tumours that in the past have been classified as embryonal sarcomas of the kidney or adult Wilms tumours would be better recognised as SSs on a molecular background [3]. Few cases (<150) of primary renal SS have been published in the English literature. Renal SSs are far more commonly monophasic/spindle cells (approximately 90%) and often show cystic change, with cysts lined by eosinophilic flat/hobnail epithelium that have been interpreted as entrapped and dilated renal tubules [3,4,5,6,7,8,9,10,11,12,13,14,15,16,17,18,19,20,21,22,23,24,25,26,27,28,29,30,31,32,33,34,35,36,37,38,39,40,41,42,43,44,45,46,47,48,49,50,51,52,53,54,55,56,57,58,59,60,61,62,63,64,65,66,67,68,69,70,71,72,73,74,75,76,77,78]. Myxoid change, when present, is described as focal and only sporadically extensive [25]. Among those cases with reported immunohistochemical results for epithelial markers (EMA and/or CKs), the vast majority (88% approximately) showed at least focal expression of one epithelial marker (Table 1). Relevant clinico-pathological data about all published cases of renal SS are summarised in Table 1.

In our case, due to the predominant cystic/myxoid appearance and location in the kidney, despite the atypical age range of our patient, CCSK was considered in the differential diagnosis. Similar to sarcomas with *BCOR* genetic alterations of bone and soft tissue, CCSK typically affects children (mean age at diagnosis: 3 years) and is characterised by ovoid cells in a myxoid background, showing significant morphological overlap with SS [2,94]. Interestingly, CCSK usually involves the renal medulla. The molecular hallmarks of CCSK are an in-frame internal tandem duplication of the *BCOR* gene, a *YWHAE::NUTM2* gene fusion or a *BCOR::CCNB3* gene fusion, all resulting in an oncogenic upregulation of the transcription factor *BCOR* [2,94].

*BCOR* (BCL6 Corepressor) gene encodes for a nuclear protein and transcription factor with a role in lymphoid development, embryonic and mesenchymal stem cell regulation, and haematopoiesis [95]. It is constitutionally expressed in the haematopoietic and lymphoid systems [96]. Somatic *BCOR* mutations were first identified in patients with acute myeloid leukaemia and have since then been reported in other haematological malignancies [97]. Apart from CCSK, *BCOR* internal tandem duplications or *BCOR* gene fusions are molecular hallmarks of a subset of high-grade central nervous system neuroepithelial tumours (CNS HGNET-BCOR) [98] and of a subset of undifferentiated soft tissue round cell sarcomas [99]. CNS HGNET-BCOR are defined by the presence of internal tandem duplications of *BCOR* [98]. They predominantly affect children, predominantly occur in supratentorial locations and are characterised by a dismal prognosis [98]. Similarly, *BCOR*-rearranged sarcomas typically arise in children or young adults, with a striking male predominance (M:F = 4.5:1) [2]. They predominantly affect the bones, followed by soft tissues but can also occur in visceral locations [2,99]. Histologically, they show considerable overlap with both CCSK and poorly differentiated SS. *BCOR* alterations that can be detected in this group of sarcomas include *BCOR::CCNB3*, *BCOR::MAML3* and *ZC3H7B::BCOR* fusion genes as well as *BCOR* internal tandem duplications [99].

BCOR immunohistochemistry is used as a valid surrogate for the diagnosis of CCKS [99] and other *BCOR*-rearranged tumours but it is important to highlight that BCOR immunohistochemical expression is not exclusive for sarcomas with *BCOR* genetic alterations: it has also been described in SSs and in other soft tissue tumours as well and can therefore be misleading [2].

Actually, BCOR upregulation has been proposed as a common downstream pathway for SSs not only with typical *SS18::SSX* fusions but also in those with rare, atypical fusion variants, which may not be recognised by FISH studies [100].

In these cases, and in all cases with atypical histomorphological or clinical features, the use of more than one molecular method is strongly advised to correctly solve the diagnosis.

## 5. Conclusions

Primary renal SS often represents a diagnostic challenge. While focal myxoid change in SS is a frequently encountered feature, such tumours may very unusually be extensively myxoid, mimicking other soft tissue tumours such as extraskeletal myxoid chondrosarcoma or *BCOR*-rearranged sarcomas. It is now recognised that BCOR immunohistochemistry is positive in a subset of SSs, representing a major diagnostic pitfall. Molecular biology represents an essential diagnostic tool in this setting.

## Figures and Tables

**Table 1:** Clinical–pathological features of renal SSs reported in the English literature.

Authors/Year	N° Cases	Age/Sex	Histology	Epithelial Markers
Argani P et al., 2000 [3]	15	20–59 yrs M:9; F:6	All 15 cases: spindle cells	EMA+ 3/6 studied cases CK− 0/5 studied cases
Kim DH et al., 2000 [4]	2	53/M 47/M	Both cases: poorly differentiated	EMA+ focal; CK AE1/AE3 + focal EMA+ focal; CK AE1/AE3 + focal
Chen S et al., 2001 [5]	1	48/M	Monophasic/spindle	EMA+ focal; CK AE1/AE3 + focal
Koyama S et al., 2001 [6]	1	47/F	Monophasic/spindle	EMA+ focal; CK AE1/AE3 + focal
Bella AJ et al., 2002 [7]	1	24/M	Monophasic/spindle	CKs+
Dai YC et al., 2002 [8]	1	19/F	Monophasic/spindle	N/A
Vesoulis Z et al., 2003 [9]	1	38/M	Biphasic	EMA+; CK AE1/AE3+; Cam 5.2+
Moch H et al., 2003 [10]	2	47/M 56/F	Monophasic/spindle Monophasic/spindle	EMA+ focal EMA+
Chen PC et al., 2003 [11]	1	19/M	Monophasic/spindle	EMA−; CK AE1/AE3+ focal
Park SJ et al., 2004 [12]	1	32/F	Monophasic/spindle	EMA−; CK−
Jun SY et al.; 2004 [13]	3	27/F 35/F 26/M	All 3 cases: monophasic/spindle with rabdoid features	CK+ focal CK− CK−
Tornkvist M et al., 2004 [14]	1	34/F	Monophasic/spindle Poorly differentiated	EMA+, CK+
Schaal CH et al., 2004 [15]	1	27/M	Monophasic/spindle Poorly differentiated	EMA+, CK AE1/AE3+
Shao L et al., 2004 [16]	4	N/A	All 4 cases: monophasic/spindle	N/A
Shannon BA et al., 2005 [17]	1	60/M	Monophasic/spindle	CK−
Perlmutter AE et al., 2005 [18]	1	61/F	Monophasic/spindle	EMA+
Paláu L MA et al., 2007 [19]	1	71/F	Monophasic/spindle with rabdoid features	EMA+; CKs−
Drozenova et al., 2008 [20]	2	33/M 57/F	Monophasic/spindle Poorly differentiated	EMA+; CKs− EMA+; CKs−
Mirza M et al., 2008 [21]	1	17/M	Monophasic/spindle	N/A
Gabilondo F et al., 2008 [22]	1	32/F	Monophasic/spindle	EMA−; CK AE1/AE3-
Zakhary MM et al., 2008 [23]	1	52/F	Monophasic/spindle Poorly differentiated	Cam 5.2+ focal; EMA−
Chung SD et al., 2008 [24]	2	30/F 49/F	Biphasic Biphasic	EMA+ EMA+
Erturhan S et al., 2008 [25]	1	59/M	Monophasic/spindle	CK7+; CKAE1/AE3+ focal
Divetia M et al., 2008 [26]	7	15–56 yrs M:2; F:5	All 7 cases: monophasic/spindle	EMA+1/4; CK−
Dassi V et al., 2009 [27]	1	20/F	Monophasic/spindle	EMA+; CKs+ focal
Kawahara et al., 2009 [28]	1	40/F	Monophasic/spindle	CK AE1/AE3+ focal
Long JA et al., 2009 [29]	3	27/M; 32/F; 33/F	All 3 cases: biphasic	EMA+; CK AE1/AE2+
Wezel F et al., 2010 [30]	1	47/M	Biphasic	EMA+
Wang Z-H et al., 2009 [31]	4	32–48 yrs M:2; F:2	All 4 cases: monophasic/spindle	EMA+ focal (3/4); CK+ focal (3/4)
Kageyama S et al., 2010 [32]	1	67/M	Biphasic	N/A
Tan YS et al., 2010 [33]	4	N/A	N/A	N/A
Romero-Rojas AE et al., 2013 [34]	1	15/M	Poorly differentiated	N/A
Lakshmaiah KC et al., 2010 [35]	2	50/F 45/M	N/A	N/A
Kataria et al., 2010 [36]	1	52/F	N/A	N/A
Grampurohit VU et al., 2011 [37]	1	21/F	Monophasic/spindle Poorly differentiated	EMA+ focal; CK+ focal
Ozkan EE et al., 2011 [38]	1	68/F	Biphasic	EMA+ focal; CK AE1/AE3−
Karafin M et al., 2011 [39]	3	39/F 41/M 53/M	All 3 cases: monophasic/spindle	N/A
Nishida T et al., 2011 [40]	1	63/F	Monophasic/spindle	CKs−
Pitino A et al., 2011 [41]	1	67/M	Monophasic/spindle	N/A
Bakhshi et al., 2012 [42]	1	33/F	Monophasic/spindle	N/A
Lopes et al., 2013 [43]	1	19/M	Monophasic/spindle	EMA+; CK AE1/AE3+
Pereira E Silva R et al., 2013 [44]	1	17/M	Monophasic/spindle	N/A
Marković-Lipkovski J et al., 2013 [45]	1	38/M	Monophasic/spindle	EMA+
Moorthy et al., 2014 [46]	1	46/M	Biphasic	EMA+; CK AE1/AE3+ focal
Majumber et al., 2014 [47]	1	46/F	N/A	N/A
Schoolmeester JK et al., 2014 [48]	16	17–78 yrs M:9; F:7	All 16 cases: monophasic/spindle	7/16 (44%) CK AE1/AE3 + focal
Ozkanli SS et al., 2014 [49]	1	45/M	Monophasic/spindle	EMA+
Mishra S et al., 2015 [50]	1	60/M	Monophasic/spindle	EMA+
Wang Z et al., 2015 [51]	1	54/F	Monophasic/spindle	EMA+; CKs+
Vedana M et al., 2015 [52]	1	76/F	Monophasic/spindle	CK7+ focal
Lv X-F et al., 2015 [53]	5	15–43 yrs M:3; F:2	N/A	N/A
El Chediak A. et al., 2016 [54]	1	26/M	Monophasic/spindle	EMA−; CK+ focal
Radhakrishnan, V. et al., 2016 [55]	1	4/F	Monophasic/spindle	EMA+
Chandrasekaran, D. et al., 2016 [56]	1	44/M	Monophasic/spindle	EMA+; CK−
Pathrose, G et al., 2017 [57]	1	25/F	Monophasic/spindle	EMA−; CK−
Pichler, R. et al., 2017 [58]	1	20/M	Monophasic/spindle	CK7−; CK20−
Chen, W. et al., 2018 [59]	1	44/M	Monophasic/spindle	EMA+; CK8/18−; CK7−
Tranesh, G. et al., 2018 [60]	1	56/M	Monophasic/spindle	EMA+ focal; CK AE1/AE3+ focal
Puj, K.S. et al., 2018 [61]	1	17/N/A	Monophasic/spindle	N/A
Dutt, U.K. et al., 2018 [62]	1	21/M	Biphasic	CK+
Cao, Z. et al., 2018 [63]	2	74/F 49/F	Biphasic Biphasic	CK AE1/AE3 + CK AE1/AE3+ focal
Stamm, A. et al., 2019 [64]	1	43/F	Monophasic/spindle	EMA+ focal; CK AE1/AE3−
Dewana, S.K. et al., 2019 [65]	1	32/M	Monophasic/spindle	EMA+ focal; CK AE1/AE3−
Xu, R.-F. et al., 2019 [66]	1	43/M	Monophasic/spindle	N/A
Cai, H.-J. et al., 2019 [67]	1	54/M	Monophasic/spindle	EMA−; CK7+
Rose, L. et al., 2019 [68]	11	N/A	6 monophasic/spindle 4 biphasic 1 poorly differentiated	6/10 EMA and/or CK AE1/AE3+
Argani, P. et al., 2019 [69]	1	35/F	Monophasic/spindle	EMA−; CK AE1/AE3−
Kanuj, M. et al., 2020 [70]	1	2/M	Monophasic/spindle	EMA+
Zhang, B. et al., 2020 [71]	1	56/M	N/A	EMA+; CK+
Krishnappa, P. et al., 2020 [72]	1	54/M	Monophasic/spindle	N/A
Huned, D. et al., 2021 [73]	1	21/M	Monophasic/spindle	N/A
Alzahrani, I. et al., 2021 [74]	1	65/M	Monophasic/spindle	EMA−; CK AE1/AE3+
Raja, A. et al., 2022 [75]	3	N/A	N/A	N/A
Fitra, A. F. et al., 2022 [76]	1	18/M	N/A	EMA+
Guimarães, T. et al., 2023 [77]	1	69/M	Monophasic/spindle	CK AE1/AE3+ focal; Cam 5.2+ focal
Challa, B. et al., 2023 [78]	14	17–72 yrs M:9; F:5	All 14 cases: monophasic/spindle	EMA+ 7/7 studied cases

## Data Availability

Not applicable.
